# Use of a Vertical Traction Device in the Management of an Open Abdomen: A Case Report

**DOI:** 10.7759/cureus.92300

**Published:** 2025-09-14

**Authors:** Rosa Miranda Thais, Aditya Benjamin

**Affiliations:** 1 Trauma and Acute Care Surgery, Liverpool Hospital, Liverpool, AUS

**Keywords:** abdominal wall surgery, fasciotens, laparostomy, open abdomen surgery, vertical traction device

## Abstract

A laparostomy procedure, a critical intervention frequently employed in both trauma and non-trauma patients, is a key component of damage control resuscitation. The use of a vertical traction device (VTD), specifically Fasciotens® Abdomen (FTA), is a relatively novel technology that prevents fascial retraction and facilitates primary fascial closure. This report describes the case of a male in his 20s with severe pancreatitis, complicated by multi-organ failure and abdominal compartment syndrome (ACS), necessitating decompressive laparotomy. FTA was applied alongside botulinum toxin (Botox) and negative pressure wound therapy (NPWT), achieving definitive closure within 10 days. Fascial distance was reduced by 8 cm within 48 hours of FTA application. This case highlights FTA’s efficacy and safety, supported by the Liverpool Hospital Trauma Acute Care Surgery (TACS) wound bundle.

## Introduction

Open abdomen is a lifesaving procedure, particularly in those with deranged physiology or critical illness. Indications commonly include trauma patients presenting with the lethal triad of hypothermia, acidosis, and coagulopathy, as well as patients with abdominal compartment syndrome (ACS), multiorgan failure, and intra-abdominal sepsis without source control.

The fascial edges of the abdominal wall are left unapproximated for purposes of re-exploration or to prevent ACS. However, leaving the abdomen open increases the risk of complications such as entero-atmospheric fistulas, frozen abdomen, and intra-abdominal abscesses [1]. Consequently, prompt closure should be considered when the patient’s physiology allows it. There are multiple temporary abdominal closure techniques, including the Wittmann Patch, Bogota Bag, negative pressure wound therapy (NPWT), and vertical traction device (VTD), which has demonstrated successful results in patients requiring abdominal wall closure.

The use of Fasciotens® Abdomen (FTA) is a novel technique that provides continuous vertical traction directly to the abdominal wall fascia, without skin tension. It also allows dynamic adjustment, with progressive fascial tension leading to re-approximation of the fascia without tearing the tissue. However, the use of FTA in Australia remains limited due to a small number of available studies in which FTA has been applied [2]. This case explores the role of FTA in a patient with pancreatitis-induced ACS, emphasizing its integration with botulinum toxin (Botox) and NPWT within a standardized protocol to enhance closure rates and reduce complications.

## Case presentation

A male in his 20s with familial hypertriglyceridemia, type 2 diabetes mellitus, and recurrent acute pancreatitis presented to the Emergency Department with sudden, severe epigastric pain radiating to both upper quadrants, accompanied by nausea and vomiting. Examination revealed a heart rate of 145 bpm, normal vital signs otherwise, and a tender epigastrium with generalized guarding.

Laboratory parameters showed leucocytosis (18.00 × 10^9^/L), lipase 600 U/L, lactate levels 7.2 mmol/L, triglycerides 79.1 mmol/L, and calcium 1.79 mmol/L. A summary of the results can be seen in Table 1.

**Table 1:** Comparison between normal laboratory values with patient values.

Test name	Normal values	Patient values
White cell count	3.5-11 x 10^9^/L	18.00 x 10^9^/L
Lipase	10-60 U/L	600 U/L
Lactate	<2 mmol/L	7.2 mmol/L
Triglycerides	<2 mmol/L	79.1 mmol/L
Calcium levels	2.10-2.60 mmol/L	1.79 mmol/L

**Figure 1:**
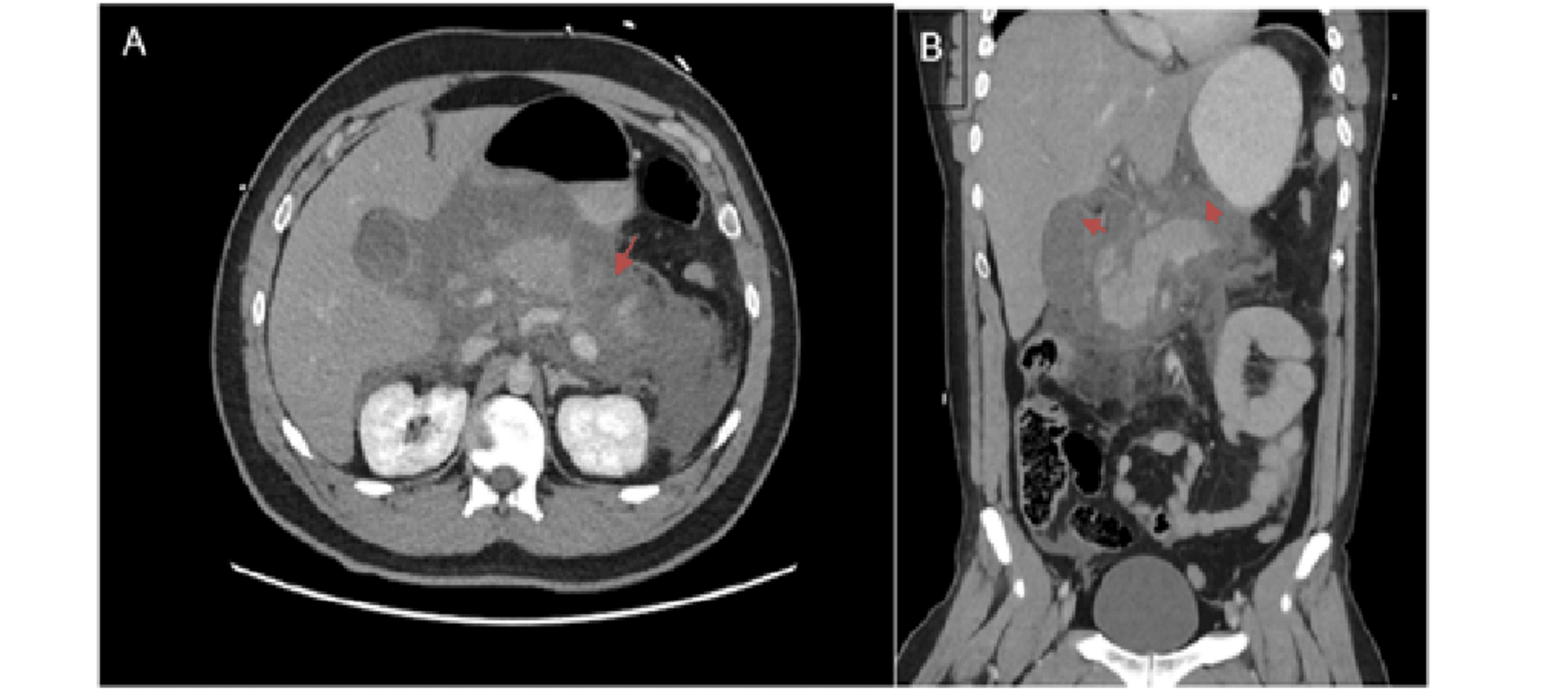
(A) Axial view showing moderate free fluid surrounding the pancreas (arrow). (B) Free fluid in the lesser sac (arrows).

A computed tomography (CT) of the abdomen and pelvis confirmed interstitial edematous pancreatitis, without evidence of necrosis or vascular complication (Figure 1).

The patient was diagnosed with severe acute pancreatitis secondary to severe hypertriglyceridemia. He received intravenous fluids, opioid analgesia, insulin-dextrose infusion, calcium gluconate, and plasmapheresis, followed by intensive care unit (ICU) transfer for monitoring.

**Figure 2:**
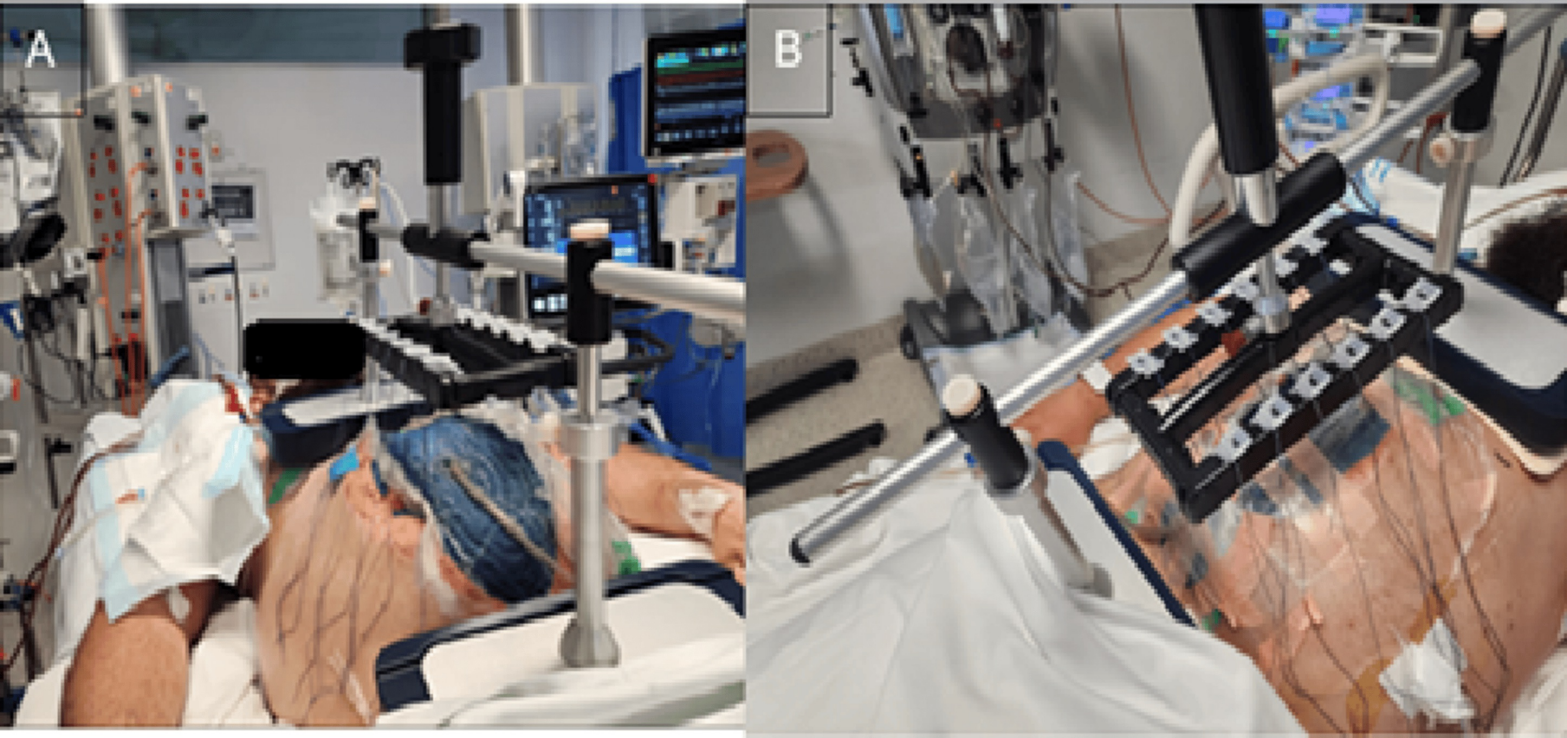
(A) Fasciotens® Abdomen scaffolding placed on the thorax and anterior pelvic ring; abdominal wall covered with Abthera (Solventum Inc.) and negative pressure wound therapy. (B) Looped sutures secured in clamps located in the retention frame.

On day 2 of admission, the patient developed multi-organ failure, requiring respiratory support and dialysis. He was diagnosed with acute compartment syndrome and underwent an urgent decompressive laparotomy and washout. Operative findings showed distal ileal necrosis, which warranted a distal ileal resection, and he was placed on NPWT with Abthera (Solventum Inc., Maplewood, MN, USA). On day 4, Botox was injected under ultrasound guidance into the abdominal wall muscles (300 units total, 150 units per side) to reduce fascial tension. Later that day, he underwent a re-look laparotomy, and this time, FTA was applied (Figure 2).

The initial fascial-to-fascial (FTF) distance was 16 cm, reducing to 8 cm within 48 hours under 6-8 units of traction (approximately 60-80 N). The FTF distance was measured during maximal traction, before and after the application of the VTD.

On day 6, he underwent a third re-look laparotomy with washout, and on day 9, definitive closure was achieved using the Trauma Acute Care Surgery (TACS) Liverpool Hospital wound bundle (small-bite closure with one polydioxanone suture (PDS), wound wash, 3-0 Monocryl skin closure, and transversus abdominis plane (TAP) catheters with ropivacaine). The patient received further supportive care for pancreatitis. He required a tracheostomy for prolonged ventilation and was subsequently weaned off dialysis and discharged home in a stable condition.

## Discussion

Laparostomy allows the restoration of physiologic stability in critically ill patients. Prompt optimization of the patient - including correction of coagulopathy, acidosis, and infection control - is essential to promote early abdominal closure. The combination of dynamic vertical traction, specifically FTA, Botox, and NPWT has been shown to decrease fascial tension and allow primary fascial closure, with a median time of 10 days and no short-term complications. However, patients with an open abdomen are at risk of complications, including fistula formation and intra-abdominal infections. Delay in fascial closure leads to higher complication rates and increases the mortality rate to up to 40% [2,3].

Abdominal wall closure in critically ill patients can be challenging, due to underlying hemodynamic instability and high mortality risk. Most patients undergo a laparostomy as part of damage control surgery, until medical stability is achieved.

Our level 1 trauma hospital follows a protocol that involves fascial traction through mesh-mediated traction using FTA, which has shown promising results; this protocol is presented in Table 2. Botox is injected under ultrasound guidance into the abdominal wall muscles (300 units total, 150 units per side) to reduce fascial tension. 

**Table 2:** Trauma and acute care surgery open abdomen protocol. NPWT: Negative pressure wound therapy; TAP: Transversus abdominis plane; Botox, Botulinum toxin; PDS, Polydioxanone suture

Step	Details
Initial Laparotomy	Damage control exploratory laparotomy, ileal resection, washout, NPWT with Abthera (Solentum Inc.)
Botox Injection	Day 4, 300 units total, 24 hours pre-Fasciotens® Abdomen
Fasciotens® Abdomen Application	Day 4, 6-8 units traction, 5 hours on/1 hour off cycle
Definitive Closure	Day 9, small-bite 1 PDS, wound wash, 3-0 Monocryl, TAP catheters with ropivacaine infusion

FTA application involves suturing a strip of Vicryl mesh to each of the fascial margins, with six Ethibond sutures evenly distributed at 6-8 units (60-80 N), adjusted as needed by ICU nurses to sustain tension. These sutures are clamped into a retention frame with cushioned support bases over the thorax and the anterior pelvic ring to distribute the weight evenly, allowing early abdominal closure (Figure 2).

The traction is sustained for five hours, followed by a one-hour rest cycle for care. Definitive closure was performed on day 9 using the Trauma and Acute Care Surgery (TACS) bundle, as previously described in the case presentation, to enhance wound integrity with local anesthesia via TAP catheters. 

The timing of fascial traction depends on the hemodynamic stability of the patient. A study from Germany applied FTA four days after damage control laparotomy. The fascial distance was measured prior to and after the application of the FTA, and a reduction of the FTF distance of 5-10 cm was found [3]. Similarly, Fung et al. reported a reduction of 5 cm in FTF distance after 48 hours of applying the VTD [4].

Botox, commonly used in elective abdominal wall reconstruction, has been shown to increase the rate of fascial closure. A retrospective study of 13 patients undergoing complex abdominal wall reconstruction found that Botox increased the rate of fascial closure, avoiding the component separation technique in 75% of cases; they found the elongation of the lateral abdominal wall muscles was up to 4.7 cm per side, with a duration of the therapeutic effect of 2-12 weeks [5].

A systematic review of the use of Botox in the emergency setting found that partial paralysis occurs from 24 hours after injection, with maximal effect occurring between 7 and 14 days. The rate of definitive fascial closure (DFC) was 90.7%, and the median time for fascial closure was five days. The study found no significant complications directly attributed to the use of Botox, although the quality of evidence was low due to the small number of trials and high levels of bias between trials [6].

Most of the studies included were able to achieve primary fascial closure between one and two weeks; the use of a VTD helps achieve closure without the need for component separation. Fung et al. reported that VTD application was associated with a mean time of seven days for definitive closure, with a range of 3-24 days, in combination with a Bogota bag and NPWT [4]. In a small retrospective study, DFC was achieved in nine patients between 5 and 14 days in combination with NPWT [3].

In our study, the mean period for VTD was six days, and DFC was achieved in 10 days without component separation. The use of VTD in combination with NPWT and Botox contributed to the success of the closure. The World Journal of Emergency Surgery guidelines (WJES) recommend continuous fascial traction combined with NPWT as the preferred technique for temporary abdominal closure, and this has been supported by Fung et al., who found a significant reduction in the FTF distance after 48 hours of applying the VTD [4]. Additionally, the Association of Coloproctology of Great Britain and Ireland supports the use of mesh-mediated traction in combination with NPWT as part of the initial management of open abdomen [6].

NPWT is known to enhance tissue perfusion, oxygenation, and the formation of granulation tissue [7]. A systematic review of temporary abdominal closure systems found that the Wittmann patch and VAC system had the highest closure rates compared to patients treated with mesh, packing, zipper, Bogota bag, and locking device [2]. In terms of intra-abdominal pressure (IAP), Dohmen et al. found a decrease from 12 to 8 mmHg after the application of VTD, with no significant impact on physiological changes such as the need for dialysis or invasive mechanical ventilation [3]. For patients at risk of ACS, the WJES guidelines recommend monitoring IAP every 12 hours and adjusting to every four to six hours once ACS is detected or if there are signs of organ failure [8].

Common complications in patients undergoing abdominal reconstruction with mesh include enterocutaneous fistula (4.5%) and incisional hernia (7%) [9]. A retrospective study performed by de Jong et al. found that only 2 out of 20 patients developed wound dehiscence and later experienced incisional hernia [5]. In a smaller study of nine patients, skin irritation and blisters were the most common complications related to VTD use [3].

Mortality rates in open abdomen patients treated with VTD vary. In a study of nine patients, three patients died before DFC was completed. In contrast, a study of 20 patients showed no mortality after VTD treatment [3,4].

The management of open abdomen requires a multidisciplinary approach; patients with open abdomen need close monitoring of their fluid balance and physiologic optimization to allow early abdominal closure. Early nutrition is crucial to prevent malnutrition; parenteral nutrition should be started as soon as possible, and enteral nutrition should be considered once resuscitation is nearly complete and the gastrointestinal (GI) tract is viable. Proper training of medical and nursing staff in the use of the VTD and monitoring of the IAP is essential for the successful management of these patients [1,8]. Our institution employs a standardized open abdomen protocol, which ensures that all staff are trained and provided with clear guidelines for managing patients with open abdomen and VTD.

## Conclusions

Open abdomen is essential for stabilizing critically ill patients, but it increases the risk of complications, including fistulas, intra-abdominal abscesses, and hernias, the longer the abdomen stays open. A multidisciplinary approach is crucial for patient optimization to allow early abdominal closure. We found that FTA prevents fascial retraction, reducing the FTF distance (e.g., 8 cm in 48 hours), facilitating early closure without component separation. However, larger and long-term studies are needed to assess further outcomes.
